# An ODeL-based teacher professional development framework for teacher-facilitated human–AI collaboration in primary mathematics problem-solving: a conceptual analysis

**DOI:** 10.3389/frai.2026.1970792

**Published:** 2026-10-23

**Authors:** Zanele Dibane

**Affiliations:** College of Education, Institute for Open and Distance Learning, University of South Africa, Pretoria, South Africa

**Keywords:** human–AI collaboration, intelligent tutoring systems, Intelligent-TPACK, mathematics problem-solving, open distance e-learning, primary education, teacher orchestration, teacher professional development

## Abstract

Artificial intelligence (AI) tools are increasingly present in primary mathematics classrooms, yet their educational value is widely argued to depend on how teachers use them. Existing models explain parts of this problem: AI-TPACK describes the knowledge teachers need, teacher-in-the-loop and human-centred AI models describe how control is shared between teachers and systems, and orchestration research describes how teachers coordinate classroom activity. What remains under-theorised is the pathway linking the way open distance e-learning (ODeL) professional development builds teachers’ capacity to its enactment when AI scaffolds learners’ problem-solving. This conceptual article asks: through which mechanisms, and under which conditions, can ODeL-based professional development be expected to enable teacher-facilitated human–AI collaboration that supports primary learners’ mathematical problem-solving? The analysis combined an integrative review of peer-reviewed and selected grey literature with theory synthesis: sources were identified through structured database searches, screened against pre-specified criteria, coded for constructs, mechanisms and boundary conditions, and organised by level of analysis. The primary context is South African primary schooling (Grades 1–7) and low-bandwidth, predominantly asynchronous ODeL. Positioning four perspectives within a multilevel meta-model, Intelligent-TPACK at the teacher level, Zone of Proximal Development scaffolding and social constructivism at the interaction and classroom levels, and Polya’s heuristic as an analytic lens at the learner level, with transactional distance theory at the programme level, I propose a three-layer framework that distinguishes inputs, mechanisms, practices and outcomes, and derive seven testable propositions. These include teacher orchestration as a mediator between AI-TPACK and scaffolding quality; infrastructure readiness, learner age, language, and AI type as moderators; and governance and children’s rights as cross-cutting boundary conditions. Unlike models that specify teacher knowledge or the division of control alone, the framework specifies the professional development pathway through which human–AI collaboration is expected to benefit learners. I propose that well-designed ODeL professional development is an enabling condition for such benefit, not a guarantee of it, and close with implementation and evaluation indicators for teacher educators, policymakers, school leaders, researchers and learners.

## 1 Introduction

The rapid expansion and dissemination of artificial intelligence (AI) throughout social, economic, and educational spheres have transformed the essence of teaching and learning in the twenty-first century. Kasneci et al. (2023) and Hwang and Tu (2021) suggest that innovative educational tools utilising artificial intelligence in education, including intelligent tutoring systems, adaptive learning platforms, and conversational AI chatbots, can enhance mathematics instruction by offering personalised feedback and tailored problem-solving pathways at a scale individual teachers cannot readily provide, although Kasneci et al. (2023) address large language models across educational levels and Hwang and Tu (2021) map research trends rather than test effects. Opesemowo and Ndlovu (2024) assert that traditional, uniform teaching methods have historically failed to address the diverse pathways through which learners develop mathematical understanding, which are influenced by their prior knowledge, cultural background, and language.

The more consequential question for this article, though, is not whether these tools can adapt to learners, but whether teachers are equipped to collaborate effectively with them. Hamedi et al. (2025) argue, in an integrative review, that AI systems can support Vygotsky’s Zone of Proximal Development (ZPD) by promptly identifying misconceptions, adjusting task complexity, and encouraging self-regulation to foster learning. Hwang and Tu’s (2021) bibliometric review likewise identifies adaptive support as a dominant focus of AI research in mathematics education. But Celik (2023) and Ng et al. (2023) emphasise a crucial point: the potential of such tools is unlikely to be realised unless teachers can integrate them purposefully into their instruction. The technology does not operate autonomously. Evidence on what happens when teachers are unprepared is, however, largely indirect for primary mathematics: much of it comes from higher education (Crompton and Burke, 2023) and secondary settings (Bastani et al., 2025) and is used here by analogy rather than as direct evidence.

Four bodies of work already explain parts of this problem. First, AI-TPACK research has extended Mishra and Koehler’s (2006) framework to AI, describing and measuring the knowledge teachers need to integrate AI-based tools ethically (Celik, 2023; Mishra et al., 2023). Second, research on classroom orchestration and teacher–AI complementarity has shown how teachers coordinate activity across individual, group and whole-class planes, and how real-time teacher tools can divide adaptive work between teachers and systems (Dillenbourg, 2013; Holstein and Aleven, 2022). Third, human-centred and hybrid-intelligence perspectives argue that AI in education should augment rather than replace human control, keeping teachers “in the loop” (EdTech Hub, 2025; Molenaar, 2022; Shneiderman, 2020). Fourth, research on ODeL and online professional development suggests that distance modes can reach rural and under-resourced teachers, provided programmes are sustained and practice-based (Darling-Hammond et al., 2017; Mhlongo et al., 2024; Thiele and Bogdon, 2022).

What these literatures do not yet explain is how they connect. AI-TPACK models describe teachers’ knowledge but not the professional development mechanisms through which it grows at a distance, nor how it is converted into moment-to-moment scaffolding decisions. Orchestration and teacher-in-the-loop models describe how control is shared in the classroom but generally assume a teacher who already has the requisite capacity and has been developed mainly in well-resourced settings with older learners. ODeL research, in turn, rarely specifies how programme design features dialogue, structure, mentoring, and community would be expected to change AI-related teaching practice. The relationships between programme design, teacher capacity, orchestration, scaffolding quality and young learners’ problem-solving, and the conditions under which those relationships hold, therefore, remain underexplored.

This article addresses the following conceptual research question: *Through which mechanisms, and under which boundary conditions, can ODeL-based teacher professional development be expected to enable teacher-facilitated human–AI collaboration that supports primary learners’ mathematical problem-solving?* Its theoretical contribution is threefold: (a) a multilevel meta-model that assigns distinct functions to the ZPD, social constructivism, Intelligent-TPACK and Polya’s heuristic, and adds transactional distance theory at the programme level; (b) a construct-specified framework that separates inputs, mechanisms, practices and outcomes and treats governance as a cross-cutting boundary condition; and (c) seven propositions, including mediating and moderating relationships, that make the framework testable and falsifiable.

Preparing teachers for this role is not a once-off training event. Evaluations of online professional development for rural middle-school science teachers (Moore et al., 2024) and studies of South African mathematics teachers’ use of online resources for professional learning (Mhlongo et al., 2024) suggest that distance modes can provide continuous, practice-integrated learning for teachers in remote or resource-constrained locations; the former is applied here to primary mathematics by analogy. In this article, ODeL is the mode of teacher professional development, not the learning environment of the learners: teachers learn through ODeL and apply that learning in face-to-face primary mathematics classrooms in which learners interact with AI tools under teacher supervision.

Scope: Three delimitations apply. First, primary learners refer to learners in Grades 1–7 (approximately 6–13 years), the span of South African primary schooling across the Foundation Phase, the Intermediate Phase and the first year of the Senior Phase. Because direct use of conversational AI raises distinct developmental and safeguarding concerns for younger children, the framework assumes teacher-mediated rather than independent chatbot use in the Foundation Phase (Grades 1–3). Second, ODeL refers to predominantly asynchronous, low-bandwidth, mobile-accessible professional development with periodic synchronous sessions and school-based practice tasks, reflecting the delivery model of open distance institutions in the region; fully face-to-face and blended modes are treated as comparison conditions (Section 4.5). Third, the primary context informing the analysis is South Africa, where multilingual classrooms, a change in the language of learning and teaching in the Intermediate Phase, and uneven connectivity and electricity supply shape both teachers’ participation and learners’ access (Mhlongo et al., 2024; Venkat and Spaull, 2015). Evidence from other countries is used by analogy, and transferability to other settings is treated as a question for empirical examination rather than an assumption.

Three concepts permeate all that ensues. Initially, the professional growth in ODeL and classroom practices is interrelated, rather than distinct. The knowledge that educators acquire online should be used in their mathematics classroom practice. Secondly, and this constitutes the core proposition of the study, AI ought to augment the capabilities of a proficient teacher rather than replace her functions. An educator who skillfully manages AI technologies, adjusts their settings, interprets their results, and determines when to intervene is engaging in a fundamentally different practice than one who has merely received a software application. Third, the orchestration role can be acquired, and I propose that ODeL is a viable method for imparting this skill, provided the program design adequately addresses the limitations of remote learning and resource-constrained environments.

## 2 Conceptual analysis method

### 2.1 Design

This article is a conceptual paper of the theory-synthesis type (Jaakkola, 2020), supported by an integrative literature review (Whittemore and Knafl, 2005). Theory synthesis was chosen because the aim is to integrate constructs from several theoretical perspectives into a new, testable account rather than to estimate effect sizes; an integrative review was chosen because the relevant evidence spans empirical, theoretical and policy sources that a systematic review restricted to a single study design would exclude.

### 2.2 Sources and search strategy

Searches were conducted in Scopus and Google Scholar and supplemented with SciSpace, an AI-assisted discovery tool, as well as backwards and forward citation tracking of key sources. Search strings combined four concept blocks: (1) artificial intelligence (“artificial intelligence” OR “intelligent tutoring system” OR “adaptive learning” OR “chatbot” OR “generative AI”); (2) mathematics problem-solving (“mathematic” OR “problem-solving” OR “word problem”); (3) primary schooling (“primary school” OR elementary OR “foundation phase” OR “intermediate phase”); and (4) teachers and professional development (“teacher professional development” OR “in-service” OR “open distance” OR ODeL OR “online professional development” OR TPACK). Searches covered January 2016 to March 2026; seminal theoretical works (e.g., Moore, 1993; Polya, 1945; Vygotsky, 1978) were included irrespective of date. Because SciSpace results are generated with the help of a language model and may be incomplete or inaccurate, every source it surfaced was in its original published form, read in full and checked bibliographically before inclusion; SciSpace was not used to screen, appraise or synthesise the literature.

### 2.3 Inclusion and exclusion criteria

Sources were included if they (a) addressed AI tools in education, teachers’ AI-related knowledge or professional development, or mathematical problem-solving; (b) concerned primary schooling or teacher professional development, or supplied a theoretical construct used in the framework; and (c) were published in English. Sources were excluded if they treated AI solely as an administrative or research tool, provided insufficient methodological or conceptual detail to judge their claims, or were commercial or promotional material.

### 2.4 Types of evidence and appraisal

Sources were classified as (i) direct evidence (empirical studies in primary mathematics), (ii) analogical evidence (empirical studies from other educational levels or subjects), (iii) theoretical or conceptual works, and (iv) grey literature (policy documents, reports and blog posts). Relevance was judged against the research question. Quality was appraised using criteria suited to each type: transparency of design, sampling, and analysis for empirical studies; conceptual clarity and internal coherence for theoretical works; and the authority, recency, and independence of the issuing body for grey literature. Appendix A reports this classification for each source cited in support of a substantive claim. Claims that rest only on analogical or grey sources are phrased conditionally in the text. Where evidence from other educational phases is used for example, higher education (Crompton and Burke, 2023), large language models across educational levels (Kasneci et al., 2023) or tutoring studies with mainly older learners (VanLehn, 2011) it is transferred only for mechanisms that are not plausibly age-specific, such as the dependence of tutoring effects on the granularity of feedback or the risk that unguided generative AI substitutes for learners’ own thinking. It is not used for effect sizes or for claims about learners’ capacities, which are expected to differ for primary learners; for these, primary- or K–12-specific evidence is used where it exists (Dignath et al., 2008; Polater and Çelik, 2026; Steenbergen-Hu and Cooper, 2013; Whitebread et al., 2009).

### 2.5 Synthesis procedure

Synthesis proceeded in four steps. (1) Extraction: from each included source, the author extracted the constructs, proposed mechanisms, outcomes and contextual conditions it described. (2) Coding: these were coded deductively against the four theoretical perspectives and inductively for mechanisms and conditions not captured by them, such as transactional distance, the provenance of feedback and governance. (3) Levelling: codes were assigned to a level of analysis programme, teacher, interaction or classroom, and learner and to a construct type input, mechanism, practice, outcome, moderator or boundary condition. (4) Relating and testing: relationships between constructs were formulated as propositions, and each was checked against disconfirming evidence and alternative explanations before being retained. The three layers of the framework are the product of step 3: they group constructs by level of analysis and construct type, not by theoretical source.

Search yield: number of records identified Scopus 87 and Google Scholar 39, screened 57 and included 23 studies. A further eight sources were added through targeted searches during revision, to incorporate meta-analytic evidence on intelligent tutoring systems in schools (Kulik and Fletcher, 2016; Ma et al., 2014; Steenbergen-Hu and Cooper, 2013), research on the development of self-regulated learning (Dignath et al., 2008; Whitebread et al., 2009; Winne and Hadwin, 1998; Zimmerman, 2002) and work on human–AI complementarity (Holstein and Aleven, 2022). Because an integrative review of this kind is vulnerable to selection and confirmation bias, studies reporting null or negative effects of AI use were deliberately sought (Bastani et al., 2025), and the evidence-type classification in Appendix A is provided so that readers can judge the basis of each claim.

## 3 Empirical background

### 3.1 Teacher professional development for AI integration in ODeL

The successful integration of AI into primary school mathematics classrooms is not a spontaneous occurrence; it requires intentional teacher preparation. A recurring argument in this literature, developed mostly in higher education (Crompton and Burke, 2023) and in ethics-focused work (Holmes et al., 2022) rather than being tested in primary mathematics, is that whether AI helps or hinders learning depends substantially on the teacher’s professional competence, not only on the tool’s sophistication. This section looks at why that competence matters, what ODeL can and cannot do to build it, and how AI literacy and human–AI collaboration function as professional skills.

#### 3.1.1 Why teachers need AI competencies

The integration of AI tools in primary mathematics classrooms imposes complex new requirements on teachers that surpass mere technological competence. Celik (2023) and Ng et al. (2023) argue that teachers need at least a working understanding of how these systems function, the logic of machine-learning algorithms, adaptive pathways, and data-driven feedback, because without it, they cannot judge when an AI output is wrong, inappropriate, or simply not helpful for a particular child. In primary mathematics, this implies a distinctly human role: reading AI-generated learning statistics and attending to the social and emotional dimensions of learning that current systems do not capture. Practically, this means translating dashboard data into decisions about grouping, sequencing, and intervention and, per Cukurova and Miao (2024), making ethical judgment calls about data privacy and algorithmic bias along the way.

Direct evidence on primary teachers’ AI literacy remains limited. Ng et al. (2023) and Celik (2023) report on teachers’ AI competencies, but with samples not specific to primary mathematics. Li (2025), in a study of primary mathematics teachers, provides the most direct evidence cited here, showing that both internal factors, such as teachers’ knowledge and beliefs, and external factors, such as school support, shape their adoption of AI. The premise that many primary mathematics teachers lack the pedagogical knowledge to weave AI tools into curriculum-aligned, learner-responsive teaching is therefore considered plausible but warrants further empirical confirmation. The risk runs in both directions: without preparation, teachers may lean on AI too heavily, effectively outsourcing instructional decisions, or avoid it altogether, forfeiting its potential benefits. EdTech Hub (2025), in a practitioner-oriented blog post summarising an expert panel, argues that both failure modes may damage learner outcomes; peer-reviewed, though analogical, evidence for the over-reliance failure mode comes from a field experiment in secondary mathematics (Bastani et al., 2025).

#### 3.1.2 ODeL as a vehicle for continuous professional development

Thiele and Bogdon (2022) describe virtual professional learning networks as one of the few realistic channels for reaching teachers who cannot attend face-to-face programmes, whether because of geography, cost, or competing professional demands. Moore et al. (2024), evaluating online professional development for rural middle-school science teachers, report evidence of its effectiveness; for primary mathematics this is analogical evidence, and the scale and quality of ODeL provision remain to be demonstrated in this context.

The features associated with effective professional development are relatively well established, although mostly from studies of face-to-face and blended programmes: sustained engagement rather than one-off workshops, practice-oriented tasks tied to teachers’ actual classrooms, collaborative online communities that support peer sensemaking, and ongoing mentoring and feedback (Darling-Hammond et al., 2017; Desimone, 2009). These features are likely to matter even more for AI-focused training, which by its nature requires cycles of learning, experimentation, reflection, and discussion, a mode of learning that a single short workshop cannot deliver. At the same time, ODeL has its own problems that programme designers cannot wish away. Faridi and Shaheen (2024) and Mhlongo et al. (2024) both report that unreliable internet, power interruptions, and inadequate infrastructure constrain teachers’ participation in resource-constrained contexts, which is precisely why offline-capable materials, asynchronous formats, and flexible assessment need to be built in from the start. How these features operate at a distance is theorised in Section 4.5.

#### 3.1.3 AI literacy, digital pedagogical competence, and human–AI collaboration

AI literacy for teachers is not one skill but several woven together: a conceptual grasp of how AI systems work, hands-on ability to use them, critical judgment about their pedagogical and ethical implications, and the reflective capacity to adjust instruction based on what the data show (Celik, 2023; Ng et al., 2021). Digital pedagogical competence, that is, using technology deliberately and analytically, rather than reflexively, underpins all of it.

Human–AI collaboration is its own professional skill, distinct from AI knowledge alone. Research on teacher AI complementarity describes it as a form of co-orchestration (Holstein et al., 2019; Molenaar, 2022) that involves orchestrating AI-mediated and teacher-led instruction into one coherent learning pathway, exercising judgement about when AI scaffolding is enough and when human intervention is needed, holding onto responsibility for the social, emotional, and ethical dimensions of learning, and continually checking whether a given tool still fits the curriculum and the learners in front of her. The premise this paper builds on is that the teacher is an active orchestrator rather than a passive user.

### 3.2 Human–AI collaboration in mathematics education

Three families of AI tools are commonly discussed in relation to primary mathematics: intelligent tutoring systems, adaptive learning platforms, and AI chatbots. Each has potential technological strengths, but in every case, it is the teacher’s orchestration that is proposed to determine whether those strengths translate into learning. Table 1 summarises what each tool does and what orchestrating it requires of a teacher. These labels describe families of products rather than mutually exclusive categories: a single system may combine an ITS’s step-level tutoring, an adaptive platform’s sequencing and a conversational interface. Ji et al. (2023) state that generative AI chatbots also differ from model-based ITSs in ways that matter for scaffolding: their outputs are stochastic, they can produce fluent but incorrect mathematics, and their knowledge is not held in an explicit, inspectable domain model. Table 2, therefore, characterises tools along six dimensions: degree of adaptivity, locus of agency, knowledge model, type of feedback, transparency and teacher control, so that the framework’s mechanisms are attached to these dimensions rather than to product labels.

**Table 1:** AI tool families in primary mathematics and the corresponding teacher orchestration role.

AI tool	What it does	What the teacher must do
Intelligent tutoring systems	Sequences practice items, gives immediate feedback, and adjusts difficulty to demonstrated skill (VanLehn, 2011; Polater and Çelik, 2026)	Select curriculum-aligned platforms, calibrate them against her own assessment of each learner, watch the progress dashboards, and step in when the automated pathway stalls
Adaptive learning platforms	Adjusts pacing and content sequencing across a topic area based on ongoing performance data (Tan et al., 2025; Xie et al., 2019)	Read the analytics to form flexible groups, spot learners who need one-to-one attention despite repeated exposure, and plan whole-class teaching around common misconceptions
AI chatbots	Holds a natural-language dialogue that can prompt Socratic questioning and metacognitive reflection (Liu et al., 2025); generative chatbots are stochastic and may produce incorrect mathematics (Ji et al., 2023)	Choose linguistically and culturally appropriate tools, set dialogue protocols that discourage answer-seeking, verify the mathematical accuracy of outputs, and review transcripts as evidence of learners’ reasoning

**Table 2:** A dimensional taxonomy of AI tools used in primary mathematics.

Dimension	Model-based ITS	Adaptive learning platform	Generative AI chatbot (LLM-based)	Implication for teacher orchestration
Degree of adaptivity	Step-level (within-task) and task-level adaptation based on a learner model (VanLehn, 2011)	Sequence-level adaptation of content, pace and pathway across a curriculum area	Turn-level conversational adaptation to learner input; usually no persistent learner model	Know the grain size at which a tool adapts and what it cannot see
Locus of agency	System-led within an author-defined problem space	System-led sequencing; teacher or learner override varies by product	Learner-initiated and open-ended	Open, learner-initiated tools need stronger dialogue protocols and supervision for young learners
Knowledge model	Explicit domain and learner models (e.g., cognitive or constraint models, knowledge tracing)	Mastery or item-response models over a curriculum map, or rule-based mastery thresholds	Statistical language model; no explicit, verifiable domain model; outputs stochastic and may be fluent but wrong (Ji et al., 2023)	Model-based errors are systematic and detectable; generative errors are unpredictable and must be verified case by case
Type of feedback	Correctness, hints and error-specific feedback at each step	Mastery and progress indicators, next-task recommendations, dashboards	Natural-language explanations, questions and, unless constrained, complete solutions	Distinguish feedback that invites reasoning from feedback that supplies answers (Table 4)
Transparency	Moderate: rules and hints inspectable by designers, rarely by teachers	Often low: proprietary algorithms summarised in dashboards	Low: reasoning not inspectable; the same prompt can yield different answers	Explainability and contestability are governance requirements (Section 7.3)
Teacher control	Configure problem sets, hint levels and pacing	Set targets, assign content, override pathways (varies)	System prompts, guardrails, access time and transcript review	Teacher control is a selection criterion, not a given

#### 3.2.1 Intelligent tutoring systems

Abinaya et al. (2024) define an intelligent tutoring system (ITS) as software that provides individualised instruction and feedback largely without direct teacher involvement in the moment. VanLehn (2011) describes these systems as approximating one-to-one tutoring, tailoring support, providing immediate feedback, and adjusting question sequences based on a learner model. Polater and Çelik’s (2026) systematic review of 24 studies of ITS use in primary school mathematics the most direct evidence base for this tool family—reports positive effects on achievement, motivation and problem-solving in many studies, but notes that results depended on learner characteristics and implementation conditions, that research was concentrated on number and operations, and that teachers needed specialised training to benefit. VanLehn’s (2011) review found that step-based tutoring systems approached the effectiveness of human tutoring, in studies drawn mainly from secondary and post-secondary settings and attributed much of this to the fine granularity of their feedback a condition that depends on accurate error diagnosis and that teacher oversight is intended to protect.

Realising those gains takes deliberate coordination: choosing a platform that fits the curriculum and the age group, calibrating it against the teacher’s own reading of each learner, watching the progress dashboards for children who are quietly falling behind, stepping in when the system’s automated scaffolding is not enough, and building collaborative, inquiry-based activity around the individual practice the ITS provides. Without this kind of coordination, I hypothesise that the use of ITS risks producing procedural fluency without the conceptual understanding that primary mathematics aims for; this is a plausible risk, consistent with the implementation dependence reported by Polater and Çelik (2026), rather than an established finding.

#### 3.2.2 Adaptive learning platforms

Tan et al. (2025) describe adaptive learning platforms (ALPs) as systems that tailor content to individual learners using adaptive technology, while Xie et al. (2019) emphasise their use of AI to adjust complexity, pacing, and pathway based on ongoing assessment. Where ITS typically walk a learner through a defined problem-solving sequence, ALPs manage personalised pathways across broader curriculum areas, something that would be practically impossible for a teacher to do by hand in a large primary class.

The value of an ALP shows up in how a teacher uses its data: grouping learners who share an error pattern for targeted teaching, identifying individuals who are still struggling despite repeated exposure and need one-to-one attention, and using platform-wide trends to plan whole-class instruction around common misconceptions. In a systematic review of 20 empirical studies spanning primary to tertiary levels, Eti et al. (2026) conclude that teachers acted as critical mediators of AI feedback and that, without such mediation, AI-driven personalisation risked increasing learner dependency. The teacher’s role as interpreter, converting algorithmic output into pedagogical decisions, is therefore proposed as a condition of the tool’s effectiveness.

#### 3.2.3 AI chatbots and conversational AI

According to Kasneci et al. (2023), AI-powered chatbots that engage in natural-language mathematical dialogue with learners are a relatively new addition to this landscape. In a design-and-evaluation study with primary students, Liu et al. (2025) report improved word-problem performance with ChatGPT-MWPS, a system built around adaptive scaffolding and contextual feedback; as a single study, this is promising but not yet generalisable evidence. Analogical evidence also shows the risk: in a field experiment in high school mathematics, Bastani et al. (2025) found that unrestricted access to a GPT-4-based tutor improved performance during practice but reduced subsequent unassisted performance, whereas a version with learning-oriented guardrails largely avoided this harm. Managing this tool well means choosing platforms that align with learners’ language and culture, designing dialogue protocols that steer conversation toward reasoning rather than answer retrieval, verifying the mathematical accuracy of its outputs, reviewing interaction logs to assess the quality of learners’ thinking, and treating those logs as formative evidence for future teaching.

Younger learners bring a particular risk to this tool. Baidoo-Anu and Owusu Ansah (2023) caution, with respect to generative AI in education generally, that users may not critically evaluate chatbot output; this concern is likely to be sharper for primary learners, whose metacognitive awareness is still developing, and an unsupervised conversational format may muddy rather than clarify their mathematical reasoning. Kapur (2016) points to the related danger of learners defaulting to answer-seeking rather than genuine problem-solving. Countering this is squarely a teacher’s job: setting explicit limits on AI use, modelling critical evaluation of AI output, and holding a standard of mathematical explanation that goes beyond anything the chatbot itself provides.

## 4 Theoretical foundations

This paper draws on four theoretical perspectives: Vygotsky’s Zone of Proximal Development, social constructivism, the Technological, Pedagogical and Content Knowledge (TPACK) framework in its Intelligent-TPACK extension, and Polya’s four-phase problem-solving model. These perspectives differ in epistemic status and level of analysis. The ZPD and social constructivism are sociocultural theories of learning and development; TPACK is a framework of teachers’ professional knowledge; and Polya’s model is a problem-solving heuristic. They are therefore not treated as equivalent foundations. Table 3 presents a meta-model that specifies, for each perspective, its epistemic status, level of analysis, principal actors, function within the framework, and relationships to the others. Transactional distance theory (Moore, 1993) is added at the programme level to explain how ODeL professional development is expected to work (Section 4.5); Section 5 then shows how the perspectives are integrated.

**Table 3:** A multilevel meta-model of the theoretical perspectives.

Perspective	Epistemic status	Level of analysis and principal actors	Function in the framework	Relationship to other perspectives
Transactional distance (M. G. Moore, 1993)	Theory of distance-education pedagogy	Programme level; programme designers, facilitators, mentors, teachers as distance learners	Explains how ODeL design—dialogue, structure, learner autonomy, supported by mentoring and community—is expected to develop teachers’ AI-TPACK (P1)	Specifies the conditions under which the teacher-level construct can develop at a distance
Intelligent-TPACK/AI-TPACK (Celik, 2023; Mishra and Koehler, 2006)	Framework of teachers’ professional knowledge	Teacher level; the teacher	Defines the capacity that professional development targets and that enables orchestration (P1, P2)	Outcome of the programme level; resource for interaction-level orchestration
Zone of Proximal Development and scaffolding (Vygotsky, 1978; van de Pol et al., 2010; Wood et al., 1976)	Sociocultural theory of learning and development	Interaction level; learner–teacher and learner–AI exchanges	Supplies criteria for scaffolding quality: contingent diagnosis, calibrated assistance, fading and transfer of responsibility (P3, P4)	Sets the standard for judging individual assistance; does not specify classroom participation structures
Social constructivism (Vygotsky, 1978; Graven and Pausigere, 2017)	Sociocultural theory of knowledge construction	Classroom level; teacher and peer groups	Specifies participation structures—discourse, collaborative problem-solving, whole-class argumentation—into which AI-supported individual work is embedded (P2)	Complements the ZPD: concerns the collective, discursive construction of meaning rather than calibration of help to an individual
Polya’s four phases (Polya, 1945), with Schoenfeld’s (1985) notion of control	Problem-solving heuristic	Learner level; the learner	Analytic lens for locating where assistance is given or withheld and for deriving observable process indicators (Table 4); not itself an outcome measure	Operationalises the learner processes at which ZPD-based scaffolding is targeted

### 4.1 Zone of proximal development

Vygotsky’s (1978) ZPD is the space between what a learner can do alone and what she can do with help from someone more capable. Within that space, systematic, well-calibrated assistance narrows the gap as the learner internalises new concepts, a dynamic that has shaped effective mathematics teaching for decades, since problem-solving so often puts learners right at the edge of what they currently understand.

Hamedi et al. (2025) analyse AI as a kind of digital scaffold within the ZPD, working through three main mechanisms: personalised learning trajectories that match content difficulty to a learner’s current level, immediate feedback that catches misconceptions before they harden, and metacognitive prompts that nudge learners toward self-regulation. On scale alone, these mechanisms give AI a potential scaffolding role: no individual teacher can personalise feedback for thirty children simultaneously in the way an adaptive system can.

But the comparison to Vygotsky’s more knowledgeable other only goes so far, and where it breaks down matters. The ZPD is inherently social and dialogic; it emerges from real, intersubjective interaction built on shared understanding, emotional connection, and cultural context (Wertsch, 1985). An AI system can process performance data, but it cannot read the social, emotional, linguistic, and cultural dimensions of a child’s ZPD the way a skilled teacher can. Hamedi et al. (2025) stress that this is exactly why the design and use of AI tools need to stay pedagogically grounded. Teachers assess a learner’s ZPD through knowledge of the whole child, not just algorithmic performance metrics, and supply the motivation, challenge, emotional support, and cultural sensitivity that AI cannot. ODeL-based professional development, then, needs to teach AI as a powerful but bounded scaffolding tool within the ZPD, not as a standalone teaching agent.

Describing AI as a “scaffold within the ZPD” nevertheless remains metaphorical unless the conditions that define scaffolding are specified. In the scaffolding literature, assistance counts as scaffolding only if it is contingent on a diagnosis of the learner’s current understanding, calibrated in amount and type, gradually faded, and oriented toward the transfer of responsibility to the learner (van de Pol et al., 2010; Wood et al., 1976). AI does not become a more knowledgeable other simply because it can supply answers or feedback. For the framework, this implies four requirements. (1) Diagnosis: AI systems estimate a learner’s position from observable indicators such as step correctness, error patterns, hint requests and response latency and, in model-based systems, knowledge-tracing estimates of skill mastery; generative chatbots typically infer it only from the current conversation.

These are proxies for performance, not direct measures of the ZPD. (2) Validation: the teacher validates the AI’s estimate by triangulating it with her own formative evidence, learners’ verbal explanations, written work and participation and overrides task assignment where the two diverge. (3) Fading: assistance is reduced when a learner succeeds on comparable tasks with less help, for example, by moving from immediate to on-request or delayed hints, withdrawing worked steps, and finally requiring an unassisted attempt and explanation; the teacher configures such fading rules where tools allow and enacts them herself where they do not. (4) Failure and uncertainty: when the AI is wrong, uncertain or out of scope, a generative chatbot producing an incorrect explanation, or an adaptive system misclassifying a learner whose difficulty is linguistic rather than mathematical, the teacher treats the output as a hypothesis to be checked, restricts or pauses the tool, and can use the error itself as a teaching opportunity. On this account, it is the teacher AI system, not the AI alone, that may function as a more knowledgeable other.

### 4.2 Social constructivism

Vygotsky’s (1978) broader claim is that knowledge is built through social interaction, dialogue, joint inquiry, and collective meaning-making rather than transmitted from teacher to learner. Graven and Pausigere (2017) studied primary mathematics, which emphasises discourse, explanation, justification, argument, and collaborative problem-solving over passive procedural drill. Mathematical understanding, on this view, is something learners build together, not something they simply receive.

Bringing AI into a classroom organised this way requires deliberate teacher coordination, because AI-driven individualisation can quietly crowd out the social learning that social constructivism treats as essential. Peer discourse and collaborative problem-solving can shrink when learners spend more time interacting with screens than with one another. The response is not to avoid AI, but to design around it, pairing AI-supported individual practice with collaborative tasks, using AI-generated performance data to form productive working groups, and running whole-class discussions that draw on what individual learners encountered in their AI interactions. The teacher, in effect, becomes the architect of the social learning environment, deciding when AI-supported individual work is appropriate and when the priority is collective mathematical talk.

Social constructivism, therefore, contributes something distinct to this framework from the ZPD. The ZPD is used at the interaction level to judge whether a given instance of assistance is contingent and fading; social constructivism is used at the classroom level to specify the participation structures, peer discourse, justification and collective argumentation within which individual AI-supported work is embedded. The two share Vygotskian roots, but they address different design questions (how much help and when, versus who talks and about what) and enter the framework at different points (scaffolding quality versus orchestration), so they are not double-counted.

### 4.3 Technological, pedagogical, and content knowledge (TPACK and Intelligent-TPACK)

Mishra and Koehler’s (2006) TPACK framework describes effective technology-integrated teaching as the intersection of technological knowledge (TK), pedagogical knowledge (PK), and content knowledge (CK). None of the three is sufficient alone; being technically skilled, knowing sound pedagogy, or having deep subject knowledge does not by itself produce good technology-integrated teaching. What matters is the integrated understanding that lets a teacher represent content in pedagogically sound ways using a particular technology. Li (2025) shows how central teachers’ knowledge is to primary teachers’ adoption of AI in mathematics instruction.

AI as an educational technology stretches the original framework. Celik (2023) proposes Intelligent-TPACK as an extension that treats the technology as an intelligent, adaptive system rather than a passive tool. Intelligent-TPACK comprises five components: intelligent technological knowledge (Intelligent-TK), intelligent technological pedagogical knowledge (Intelligent-TPK), intelligent technological content knowledge (Intelligent-TCK), their integration (Intelligent-TPACK), and ethical knowledge about AI-based tools (Ethics). It differs from the original TPACK in two respects: its technological knowledge concerns adaptive, data-driven systems whose outputs must be interpreted and verified, and it adds ethics as an explicit knowledge component. For readability, this article uses “AI-TPACK” as shorthand for Celik’s Intelligent-TPACK construct throughout. Applied to primary mathematics, Intelligent-TK covers how machine learning, natural language processing and adaptive algorithms broadly work; Intelligent-TPK covers how AI can personalise instruction, support formative assessment and differentiate learning; Intelligent-TCK covers the ability to judge AI-generated mathematical explanations and feedback for accuracy and conceptual soundness; and the ethics component covers fairness, transparency, privacy and accountability in AI use. The implication for ODeL professional development is that it needs to build all components together; treating AI competence as a technical add-on, separate from pedagogy and content, misses the point of the framework entirely.

UNESCO’s AI competency framework for teachers (Cukurova and Miao, 2024) provides a point of comparison. It specifies five dimensions: human-centred mindset, ethics of AI, AI foundations and applications, AI pedagogy, and AI for professional development across three progression levels (acquire, deepen and create). When mapped against it, AI-TPACK covers AI foundations and applications (Intelligent-TK), AI pedagogy (Intelligent-TPK, -TCK, and -TPACK), and ethics, but is weaker in a human-centred mindset and in the use of AI for teachers’ own professional learning. The framework addresses these gaps outside the knowledge construct: human-centredness through the governance boundary condition and the positioning of learners as rights holders (Sections 7.3 and 8), and AI for professional development at the ODeL programme level, where AI tools are modelled in a manner congruent with the programme itself (Section 4.5).

Development of AI-TPACK can be indicated by (a) a change in self-reported Intelligent-TPACK on Celik (2023) validated scale; (b) the quality of teachers’ AI-integrated lesson designs and of their written critiques of AI-generated mathematics, scored with a rubric; and (c) the observed quality of orchestration (Section 6). Because self-reported and enacted knowledge often diverge, the framework treats (b) and (c) as the stronger indicators.

For primary mathematics teachers specifically, content knowledge encompasses the subject matter itself, learners’ developmental trajectories, common misconceptions, and the curriculum’s demands for problem-solving. Pedagogical knowledge covers facilitating discourse, managing cooperative learning, using formative assessment, and designing tasks that invite productive struggle. Weak content or pedagogical knowledge undermines a teacher’s ability to critically evaluate AI-generated content and feedback, which is exactly why AI-TPACK professional development must address all its components, not just the technological ones (Ball et al., 2008; Venkat and Spaull, 2015).

### 4.4 Polya’s four-phase problem-solving model

Polya (1945) four-phase approach, understanding the problem, devising a plan, carrying it out, and looking back, has anchored mathematics education research and teaching for more than seventy years. The model has remained relevant because it foregrounds the metacognitive side of problem-solving: the reflective habits by which successful problem-solvers track their understanding, assess whether a strategy is working, and learn from both successes and failures.

Polya’s model is used here as an analytic lens rather than as a measure of learning outcomes, and it is not assumed to operate linearly: problem-solvers move back and forth between phases, returning to understanding when a plan fails, for example. Alternatives were considered. Schoenfeld’s (1985) framework of resources, heuristics, control and beliefs offers a richer account of metacognitive control, and research on productive failure (Kapur, 2016) specifies when withholding assistance benefits learning. Polya’s model is retained as the organising lens for three reasons: its phases are simple enough to be taught to, and used by, primary learners and their teachers; it is widely used in primary mathematics teacher education; and its phase boundaries mark the points at which AI assistance can be introduced, delayed or withheld. Schoenfeld’s notion of control and Kapur’s productive failure are drawn on to supply the metacognitive and productive-struggle criteria that Polya’s phases alone do not provide.

Polya formulated his heuristic with relatively mature problem-solvers in mind, so using it with learners aged roughly 6–13 requires developmental grounding. Models of self-regulated learning describe how learners plan, monitor and evaluate their work in cyclical phases of forethought, performance and self-reflection (Zimmerman, 2002), and how they set goals and adjust strategies against internal standards while working on a task (Winne and Hadwin, 1998). These map closely onto Polya’s phases: understanding and planning correspond to forethought; carrying out the plan corresponds to performance and monitoring; and looking back corresponds to self-reflection. Research with young children shows that metacognitive and self-regulatory behaviour appears earlier than was once assumed but is largely implicit visible in what children do and say during a task rather than in their ability to report on it (Whitebread et al., 2009) and a meta-analysis of self-regulation training in primary schools shows that such strategies can be taught effectively at this age (Dignath et al., 2008). The framework therefore scales Polya’s phases developmentally. In the Foundation Phase (Grades 1–3), the phases are enacted jointly with the teacher through talk, visual prompts and routines, and looking back is evidenced behaviourally (for example, checking an answer with concrete materials) rather than verbally. In the Intermediate Phase (Grades 4–6), learners are prompted to name the phases, choose among the offered strategies, and explain a check. By Grade 7, learners can be expected to plan and evaluate more independently, with scaffolding faded accordingly. This developmental gradient sharpens the risk identified by Baidoo-Anu and Owusu Ansah (2023): judging whether a chatbot’s explanation is correct is itself a late-developing self-evaluative skill, which is why the framework assumes teacher-mediated rather than independent chatbot use for younger learners and treats learner age as a moderator (P5).

AI tools may support each of these four phases, though in different ways. In the understanding phase, a chatbot can run a Socratic dialogue that probes a learner’s grasp of the problem’s structure and surfaces hidden assumptions, while an ITS can catch when a learner has systematically misread the problem’s conditions. In the planning phase, an ALP can offer progressively challenging analogous problems that help a learner select an effective strategy, and a chatbot can prompt relevant prior knowledge without simply handing over a solution path. In the execution phase, ITS provides step-by-step guidance and quick feedback that keeps a learner working through difficulty rather than abandoning a sound strategy. In the reflection phase, AI tools can prompt learners to review their process, suggest alternative methods, and generate similar problems for varied practice.

Table 4 derives observable indicators for each phase and distinguishes productive scaffolding from the substitution of the learner’s own thinking. The general criterion is that assistance is productive when it leaves the essential cognitive work of the phase with the learner and is faded over time; it is substitutive when it performs that work for the learner for instance, by restating the problem in solved form, choosing the strategy, executing the calculation or supplying the check.

**Table 4:** Observable indicators for Polya’s phases and criteria distinguishing productive scaffolding from substitution.

Phase (non-linear)	Observable learner indicators	Productive scaffolding (AI or teacher)	Substitution (to avoid)
Understanding the problem	Restates the problem in own words or home language; identifies knowns, unknowns and conditions; draws a representation	Prompts to restate and identify quantities; read-aloud or translation, where reading is not the learning target	Rewriting the problem as a bare calculation, naming the operation for the learner
Devising a plan	Name or select a strategy and give a reason; relate the problem to a similar one	Questions about similar problems, a choice of representations; hints only after an attempt	Supplying the strategy or solution path, showing a worked example of the same problem before an attempt
Carrying out the plan	Executes steps while self-monitoring; detects and repairs own errors	Flagging that an error exists and asking the learner to locate it; step feedback that fades	Correcting errors automatically, completing steps, and answer-checking on demand without reasoning
Looking back	Checks reasonableness; explains why the solution works; compares methods; generalises	Prompts to check and explain; comparison with peers’ methods; varied follow-up problems	AI supplies the check or the explanation; AI-generated reflection is accepted in place of the learner’s own
Across phases (dependence)	Help requested before any attempt; share of answer-seeking prompts in chatbot transcripts; gap between assisted and unassisted performance	Assistance declines across comparable tasks; unassisted attempts and explanations required periodically	Assistance remains constant or increases; learner cannot proceed without the tool

Across all four phases, though, the teacher’s role stays indispensable. AI can prompt reflection, but it cannot guarantee that a learner is genuinely engaging in it. It can suggest alternative strategies, but it cannot model expert mathematical thinking to explain why one strategy beats another. It can generate similar problems, but it cannot facilitate the collective classroom discussion in which learners articulate, compare, and refine their approaches together, a process that both Kapur (2016) and Polya (1945) treat as central to real learning. Teachers should use Polya’s model less as a fixed checklist and more as an analytic lens: a way of deciding, phase by phase, which learners need more human scaffolding and where instruction should push for reasoning and metacognition rather than settle for procedural fluency.

### 4.5 Transactional distance: a Programme-level perspective

Transactional distance is Moore (1993) term for the psychological and communicative space between learners and instructors in distance education a pedagogical rather than a geographical distance. It is theorised to vary with three interacting variables: dialogue (purposeful, constructive interaction between instructor and learner), structure (the rigidity or flexibility of a programme’s objectives, activities and assessment) and learner autonomy (the extent to which learners direct their own learning). High structure with little dialogue produces high transactional distance and demands greater autonomy from learners; more dialogue and responsive structure reduce it. Later work adds that a community of inquiry, combining social, cognitive and teaching presence, supports sustained, critical learning at a distance (Garrison et al., 2000).

Applied to AI-focused professional development, these variables suggest specific mechanisms. Dialogue with facilitators and mentors about teachers’ own classroom attempts with AI is expected to develop Intelligent-TCK and ethical judgement, because verifying AI-generated mathematics and deciding when to intervene are judgements best learned through feedback on practice. Structure sequenced cycles of learning, trying in class, reflecting and sharing is expected to support the formation of orchestration routines, but must remain flexible enough for teachers with irregular connectivity. Supported learner autonomy is expected to allow teachers to adapt AI use to their own grade, language and infrastructure. Mentoring and professional community are expected to sustain participation and to provide the collective sense-making through which teachers interpret AI analytics and share orchestration strategies (Graven and Pausigere, 2017; Thiele and Bogdon, 2022).

From these mechanisms and the professional development literature, five ODeL design principles are derived: (1) *sustained engagement* duration and spacing sufficient for repeated cycles of practice (Darling-Hammond et al., 2017; Desimone, 2009), which reduces transactional distance through ongoing dialogue; (2) *practice-based learning* tasks enacted in teachers’ own classrooms and brought back for feedback (Darling-Hammond et al., 2017; Kennedy, 2016); (3) *collaborative community* peer and mentor networks that sustain dialogue and social presence (Garrison et al., 2000; Thiele and Bogdon, 2022); (4) *contextual responsiveness* flexible structure, low-bandwidth and offline-capable materials, and multilingual resources that fit teachers’ infrastructural and linguistic conditions (Faridi and Shaheen, 2024; Mhlongo et al., 2024); and (5) *congruent modelling* the programme uses AI in the way teachers are expected to use it with learners, with facilitator mediation, verification of outputs and attention to ethics, so that teachers experience teacher-in-the-loop AI use as learners (Swennen et al., 2008). The first three principles restate the features of effective professional development reviewed in Section 3.1 (Darling-Hammond et al., 2017) sustained duration, practice orientation and collaboration, with mentoring folded into community while the last two are added because ODeL and AI, respectively, make them necessary: contextual responsiveness addresses the infrastructural conditions of distance participation, and congruent modelling addresses the need for teachers to experience the practice they are expected to enact. These five principles are derived here, before the framework, and are only applied in Sections 8 and 10.

The claim that ODeL is a suitable mode must be qualified by comparison. Face-to-face professional development permits richer dialogue and immediate modelling but is costly to sustain and difficult to provide for teachers in remote areas; blended models combine these advantages and may outperform fully distance modes where some contact is feasible. ODeL is therefore proposed not as the most effective mode in general, but as a mode that can offer sustained, practice-based development at scale, where face-to-face contact is infrequent and occurs only when teachers have sufficient connectivity, devices, electricity, time, and institutional recognition to participate. Where these conditions are absent, ODeL may widen rather than narrow inequities, and blended or school-based models may be preferable.

## 5 Integrating the theoretical perspectives

These four perspectives are not separate accounts of separate parts of the problem. They interlock, and each one illuminates something the others cannot fully capture on their own. This section pulls them together into the paper’s central theoretical proposition: an explanation of how ODeL-based teacher professional development is expected to create the conditions for human–AI collaboration that may improve primary learners’ mathematical problem-solving.

The reasoning runs roughly as follows. Effective use of AI in primary mathematics classrooms is proposed to depend substantially on teacher professional development, which in the contexts considered here is most feasibly delivered through ODeL, because this development shapes how skilfully teachers use AI tools in the first place. Teachers are unlikely to use AI well without sustained, practice-oriented development across AI literacy, pedagogical reasoning, and mathematics content knowledge. Where infrastructural and participation conditions are met, ODeL can deliver that development at scale, provided its design reduces transactional distance through dialogue, flexible structure, mentoring and community (Section 4.5) and does not create new access gaps that exclude teachers in remote or under-resourced settings.

What that development specifically builds is AI-TPACK, an integrated knowledge base that enables teachers to select, configure, and use AI tools in ways that align with the curriculum. AI-TPACK is not a fixed credential; it is a living, contextual body of knowledge that grows through cycles of learning, practice, and reflection, which is precisely what well-designed ODeL professional development is intended to deliver. Without AI-TPACK, teachers are unlikely to function as genuine facilitators of AI-enhanced instruction; with it, they can bring principled professional judgement to a human–AI partnership rather than simply deferring to the tool.

That judgement is what makes collaboration effective, rather than merely present. A teacher is collaborating with AI, in the sense this paper means, when she deploys it deliberately as a pedagogical instrument, not an autonomous instructional agent, while retaining responsibility for the social, emotional, ethical, and intellectual dimensions of her learners’ mathematical experience, and strategically using AI’s strengths in personalisation, adaptive feedback, and analytics. This is the paper’s central thesis in practice: AI extends teachers’ capabilities; it does not substitute for them. This position is well established in research on classroom orchestration and human–AI complementarity (Holstein and Aleven, 2022; Molenaar, 2022); the contribution of this article lies not in restating it but in specifying the professional development pathway through which teachers become able to act on it, and the conditions under which it holds (Section 9.1).

How teachers exercise that orchestration is grounded in both ZPD theory and social constructivism. Under ZPD, AI tools offer personalised, flexible scaffolding matched to each learner’s current level, adjusted incrementally as understanding grows, with the teacher supervising the process to account for the social, emotional, and cultural dimensions of a child’s ZPD that current algorithms do not capture. Under social constructivism, the teacher builds the wider social learning environment around AI-supported individual practice, collaborative tasks, mathematical discourse, and peer learning, supplying the social dimension of knowledge-building that AI interaction alone cannot provide.

Using AI-TPACK, the teacher then coordinates ZPD-oriented AI scaffolding and socially constructivist classroom design to support learners across every phase of Polya’s model. AI may reduce extraneous cognitive load for example, the reading demands of a word problem in a second language, or routine calculation that is not the learning target so that learners’ effort is directed at the mathematical structure of the problem (Sweller, 1988). It should not absorb the intrinsic work of understanding, planning, executing and checking, because that effortful work is where productive struggle, and therefore learning, occurs (Hiebert and Grouws, 2007; Kapur, 2016). Deciding which load is which, for a given learner and task, is part of the teacher’s orchestration. Whether pairing AI scaffolding with teacher-led metacognitive development produces better problem-solving outcomes than either alone is a central hypothesis of the framework (Proposition 4), not an established finding.

Taken together, this integration cuts through a debate that has, frankly, generated more heat than light: whether AI should replace teachers or be dismissed as an unwelcome distraction. Neither framing fits the evidence reviewed here. AI is neither a substitute for teachers nor a peripheral add-on; it is a powerful yet genuinely bounded instructional tool, and its value depends on the very human expertise it is sometimes assumed to replace. ODeL is proposed as a promising platform for building that expertise at scale where face-to-face provision is not feasible, subject to the conditions set out in Section 4.5.

## 6 Proposed conceptual framework

Pulling together the theoretical synthesis in Sections 4 and 5 with the literature reviewed in Section 3, I propose a three-layer conceptual framework for teacher-facilitated human–AI primary mathematics instruction. It is worth being explicit about what kind of framework this is: not a linear sequence of steps, but a dynamic system in which each layer both shapes and depends on the others. Figure 1 presents the framework visually; the subsections that follow unpack each layer and its interactions with the others, define each construct (Section 6.4) and specify the relationships as testable propositions (Section 6.5).

**Figure 1:**
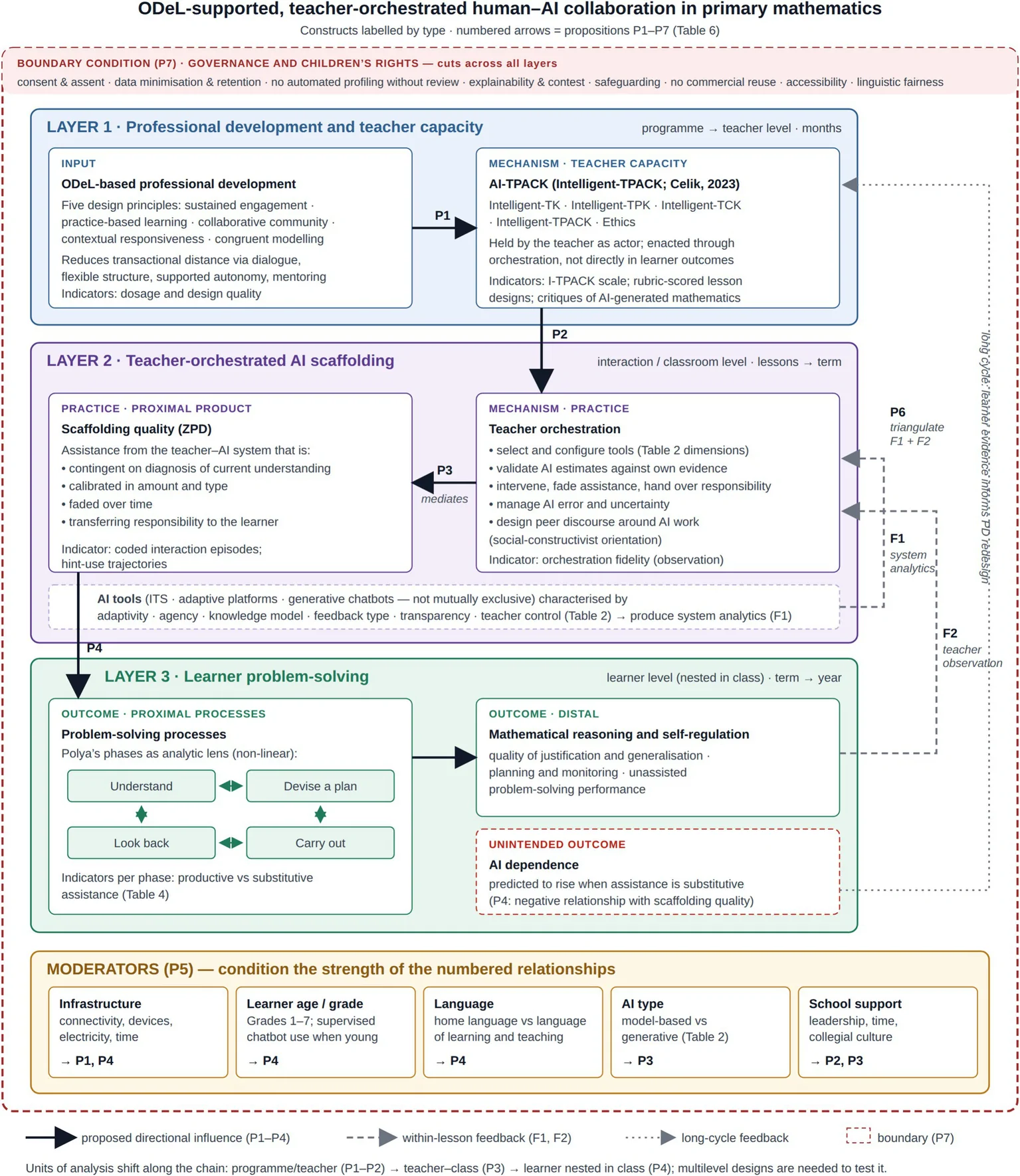
A three-layer conceptual framework of teacher-facilitated human–AI collaboration in primary mathematics, supported by ODeL-based professional development.

Constructs are labelled by type (input, mechanism, practice, outcome); numbered arrows correspond to propositions P1–P7 (Table 5). Solid arrows denote proposed directional influence; dashed arrows denote feedback, distinguishing AI system-generated analytics (F1) from teachers’ professional observations (F2). F1 carries mastery estimates, error patterns, hint use and chatbot transcripts from AI tools to the teacher in real time or daily; F2 carries the teacher’s own observations of learners’ explanations, participation and language use within and across lessons; the long-cycle loop carries teachers’ practice portfolios, with de-identified analytics summaries and reflections, to ODeL facilitators at the end of each practice cycle (every few weeks), and pooled portfolio evidence to programme designers at the end of each module or semester (Section 6). Moderators (P5) and the governance boundary condition (P7) act across layers. Synthesised from Moore (1993), Vygotsky (1978), van de Pol et al. (2010), Mishra and Koehler (2006), Celik (2023) and Polya (1945).

**Table 5:** Propositions derived from the framework.

Propositions P1-P7	Proposition	Proposed mechanism	Unit of analysis	Timescale
P1	The design quality and dosage of ODeL professional development are positively related to growth in teachers’ AI-TPACK.	Reduced transactional distance through dialogue, flexible structure, mentoring and community; repeated practice cycles	Teacher (nested in programme cohort)	Months
P2	AI-TPACK is positively related to the quality of teacher orchestration.	Knowledge enables tool selection and configuration, verification of outputs and timing of interventions	Teacher	Weeks to months after professional development
P3	Teacher orchestration mediates the relationship between AI-TPACK and scaffolding quality.	Knowledge affects learners’ experience only when enacted as validation, fading and social design	Teacher–class; lesson	Lessons to a term
P4	Scaffolding quality is positively related to learners’ problem-solving processes and reasoning and negatively related to AI dependence.	Contingent, fading assistance keeps the essential cognitive work with the learner (productive struggle)	Learner nested in class	Weeks to a term
P5	Infrastructure readiness moderates P1 and P4; AI type moderates P3 (generative tools require more verification); learner age and alignment between home language and the language of learning and teaching moderate P4; school support moderates P2 and P3.	Conditions constrain participation, access, the verification burden and the fit of assistance	Programme, class, learner	As for the moderated relationship
P6	Orchestration decisions are of higher quality when teachers triangulate system-generated analytics (F1) with professional observation (F2) than when they rely on either alone.	Each channel captures what the other misses	Teacher decisions	Within lessons
P7	Governance conditions (Section 7.3) are necessary for implementation; where they are unmet, the framework does not recommend AI use, regardless of P1–P4.	Protects children’s rights and the legitimacy of the collaboration	School and system	Before and throughout implementation

The interaction between the three layers runs in both directions, not down a single hierarchy. AI-generated learning analytics (Layer 2) feed into teachers’ ongoing professional decisions (Layer 1); teachers’ pedagogical judgements, in turn, shape how AI scaffolding is configured (Layer 2); and evidence of learners’ problem-solving development (Layer 3) flows back into both teacher practice and AI adaptation. Two feedback channels are distinguished. System-generated feedback (F1) consists of analytics produced by the AI mastery estimates, error patterns, time on task, and chatbot transcripts; it is fast and scalable but is limited to what the system records and is subject to model error. Teachers’ professional observation (F2) consists of evidence that the teacher gathers directly from learners’ explanations, participation in discussion, affect, and language use; it is slower but captures dimensions that the system cannot. The framework proposes that orchestration decisions are better when they triangulate both channels (P6).

The long-cycle loop is institutional, so its mechanism needs to be specified in concrete terms. At the end of each practice cycle in the ODeL programme (for example, every three to four weeks), teachers submit a practice portfolio to their facilitator or mentor: the lesson design, a short reflection, their own observation notes (F2) and a de-identified, class-level summary of the relevant platform analytics (F1), such as mastery and hint-use patterns or anonymised chatbot excerpts. Only aggregated or de-identified data leave the school, consistent with the governance conditions in Section 7.3. Facilitators respond within the cycle, and programme teams review pooled portfolios at the end of each module or semester and revise content, tasks and mentoring accordingly. The loops therefore run on three timescales: within lessons (F1 and F2 to the teacher), across practice cycles of a few weeks (portfolio to mentor and back), and across modules or semesters (pooled evidence to programme redesign).

### 6.1 Layer 1: professional development and teacher capacity (input and mechanism)

The first layer is the foundation on which everything else rests. It comprises teacher professional development delivered through ODeL, which builds an integrated AI-TPACK knowledge base, strengthens teachers’ professional expertise in primary mathematics instruction, and embeds social-constructivist pedagogical principles that keep AI tools folded into collaborative, discourse-rich learning rather than isolated screen time. This layer expresses the framework’s central proposition: lasting learning gains in primary mathematics through AI are proposed to depend substantially on a professionally prepared teacher.

Layer 1 contains constructs of different types that should not be conflated. ODeL-based professional development is an input, an intervention at the programme level. AI-TPACK is a mechanism, a teacher capacity developed by that input. The teacher is the actor who holds that capacity and exercises professional judgement; teacher expertise is thus not a separate construct but the capacity in use. Social constructivism is a pedagogical orientation that shapes how capacity is enacted in classroom practice and therefore operates in Layer 2 through orchestration. ODeL development is proposed to build AI-TPACK (P1), and AI-TPACK to shape orchestration (P2), which in turn shapes the learning environment in which AI scaffolding operates. None of this is a one-time achievement; teacher professional development must continue to evolve alongside advances in AI technology, curriculum changes, and shifting learner needs.

### 6.2 Layer 2: teacher-orchestrated AI scaffolding (practices)

Layer 2 separates three kinds of elements. The practice is teacher orchestration: selecting and configuring tools, validating AI estimates, deciding when to intervene or fade assistance, managing AI error, and designing the social learning around AI use. The tools (ITSs, adaptive platforms and chatbots) are characterised by the dimensions in Table 2, and their type is treated as a moderator rather than as a construct. The data and feedback produced by the tools constitute an information flow (F1), distinct from teachers’ own observations (F2). The proximal product of this layer is scaffolding quality: the extent to which learners receive contingent, calibrated, fading assistance that transfers responsibility to them (van de Pol et al., 2010). This layer sits deliberately between the pedagogical foundation (Layer 1) and learner outcomes (Layer 3) because, in this framework, AI scaffolding is not intended to be autonomous; it is mediated by teacher judgement and ultimately aimed at learners’ mathematical problem-solving. The elements within Layer 2 interact with both neighbours: AI tools generate analytics that flow up to inform teacher decision-making; teacher decisions about task design, grouping, and intervention flow down to shape the AI scaffolding environment; and AI scaffolding interacts directly with learners’ problem-solving processes in Layer 3, offering the kind of personalised, adaptive support that may help learners engage productively with each phase of Polya’s model.

### 6.3 Layer 3: learner mathematical problem-solving development (outcomes)

Layer 3 distinguishes learner processes from outcomes. Processes are the observable problem-solving behaviours in each of Polya’s phases (Table 4), which serve as proximal indicators. Outcomes are the quality of learners’ mathematical reasoning, their self-regulation, and their unassisted problem-solving performance. AI dependence is included as an unintended outcome that the framework predicts will rise when scaffolding is substitutive. These outcomes are the framework’s real test; they are proposed to depend on the interplay of all three layers, not on any one layer in isolation. Layer 3 also feeds back into Layer 1: as learners’ problem-solving develops, system-generated analytics (F1) and teacher observations (F2) become evidence that shapes further professional development and instructional decisions. The framework is deliberately not a linear pipeline; it is a responsive system in which teacher growth, AI-mediated instruction, and learner progress keep shaping one another over time.

### 6.4 Construct specification

Table 6 defines each construct, its type, level of analysis, example indicators and the timescale over which it is expected to change.

**Table 6:** Construct specification.

Construct	Type	Level	Definition	Example indicators (timescale)
ODeL-based professional development	Input	Programme	Distance professional development designed on the five principles in Section 4.5	Dosage (hours, weeks, practice cycles completed); design quality (fidelity to principles, frequency of facilitator dialogue, mentor contact) (months)
AI-TPACK (Intelligent-TPACK)	Mechanism (capacity)	Teacher	Integrated knowledge for using AI-based tools with mathematics content and pedagogy, including ethics (Celik, 2023)	Intelligent-TPACK scale; rubric-scored AI-integrated lesson designs; critiques of AI-generated solutions (months to a year)
Teacher orchestration	Mechanism (practice)	Teacher–classroom	Planned and real-time coordination of AI and human activity: select and configure, validate, intervene or fade, manage error, design social learning	Observation protocol; orchestration fidelity checklist; teacher logs (lessons to weeks)
Scaffolding quality	Practice (proximal product)	Interaction	Extent to which assistance from the teacher–AI system is contingent, calibrated, faded and transfers responsibility	Coded interaction episodes; hint-use trajectories across comparable tasks (lessons)
Problem-solving processes	Outcome (proximal)	Learner	Observable behaviour in each Polya phase	Table 4 indicators; think-alouds; chatbot transcripts (tasks to weeks)
Mathematical reasoning and self-regulation	Outcome (distal)	Learner	Quality of justification and generalisation; planning and monitoring of one’s own problem-solving	Reasoning rubric on unassisted tasks; age-appropriate self-regulation measures (term to year)
AI dependence	Outcome (unintended)	Learner	Reliance on AI that substitutes for the learner’s own thinking	Help-seeking before attempts; answer-seeking prompts; assisted–unassisted performance gap (weeks to term)
F1 system-generated feedback; F2 teacher professional observation	Information flows	Interaction to teacher	F1: analytics produced by AI tools; F2: evidence gathered directly by the teacher	Use of each source in documented decisions (continuous)
Infrastructure readiness; learner age/grade; language of learning and teaching; AI type; school support	Moderators	Programme, class, learner	Conditions that strengthen or weaken specified relationships (P5)	Connectivity, device and electricity audit; grade; home language versus language of learning and teaching; Table 2 classification; leadership support
Governance and children’s rights	Boundary condition	School and system	Legal, ethical and institutional conditions under which AI use with children is permissible (Section 7.3)	Consent and assent records; data protection impact assessment; retention policy; accessibility audit

### 6.5 Relationships and propositions

In Figure 1, each solid arrow denotes a proposed positive directional influence from the construct at its tail to the construct at its head, labelled with its proposition. The unit of analysis shifts along the chain programme and teacher (P1–P2), teacher and class (P3), and learner nested in class (P4) so multilevel designs are needed to test it. The chain is also temporally ordered: effects of professional development on AI-TPACK are expected over months, changes in orchestration and scaffolding over weeks to a term, and learner outcomes over a term or longer. Dashed arrows are feedback loops that operate within lessons (F1 and F2 informing orchestration) and over longer cycles (learner outcomes informing programme redesign). Table 5 states the propositions.

#### 6.5.1 Candidate measures

P1: ODeL platform logs (hours, practice cycles completed, dialogue posts), a design-fidelity rubric, Celik’s (2023) Intelligent-TPACK scale administered before and after the programme, and rubric-scored lesson designs. P2: the Intelligent-TPACK scale and artefacts, a classroom observation protocol with an orchestration-fidelity checklist, and teacher decision logs. P3: orchestration observations and classroom episodes coded for contingency and fading (van de Pol et al., 2010), analysed with multilevel mediation models. P4: coded scaffolding episodes; platform interaction logs (hint use, help-seeking before attempts); unassisted problem-solving tasks scored by Polya phase (Table 4); and a mathematical-reasoning rubric. P5: an infrastructure audit, learners’ grade, home language relative to the language of learning and teaching, tool classification (Table 2) and a school-support survey, entered as interaction terms in multilevel models. P6: teacher decision logs recording the source of evidence used (F1, F2 or both), stimulated-recall interviews and rated decision quality. P7: a governance audit of consent and assent records, data protection impact assessments, retention policies and accessibility reviews.

To illustrate how the propositions combine into a testable model: P1–P3 jointly imply that growth in teachers’ AI-TPACK mediates the relationship between the intensity of ODeL participation and the quality of metacognitive scaffolding observed in Polya’s looking-back phase. This could be tested in a longitudinal multilevel design that measures participation from platform logs; AI-TPACK using Celik’s (2023) instrument at the start and end of the programme; looking-back scaffolding from coded classroom observations and platform interaction logs; and learners’ unassisted problem-solving assessments as the downstream outcome (P4).

## 7 Challenges and opportunities for human–AI collaboration

Realising this framework in practice means navigating real opportunities alongside real challenges, and a theoretically honest assessment must weigh both rather than defaulting to either optimism or caution.

### 7.1 Opportunities

AI tools offer potential mechanisms for individualised learning pathways matched to each learner’s current mathematical level, something that is otherwise very hard to deliver in primary classrooms with high learner-to-teacher ratios. These are proposed mechanisms whose realisation depends on model accuracy, data quality, task design and implementation, and the evidence for each differs in type and strength. Intelligent tutoring systems and adaptive learning platforms can provide timely, individualised feedback, which may allow learners to catch and correct misunderstandings quickly (Abinaya et al., 2024; Xie et al., 2019), provided the underlying model diagnoses errors accurately. The strongest evidence for such feedback comes from reviews of step-based tutoring conducted mainly outside primary settings (VanLehn, 2011), with more limited and implementation-dependent direct evidence in primary mathematics (Polater and Çelik, 2026), thereby potentially turning each problem-solving attempt into a formative learning moment rather than a graded test. AI-generated analytics may also give teachers real-time data on individual and class-wide performance, supporting evidence-based decisions about grouping, sequencing, and intervention (Holstein et al., 2019; Kasneci et al., 2023), although the quality of those decisions depends on the validity of the underlying data. By automating routine formative assessment and tailored practice, these tools may free some teacher time for higher-order instructional work, such as mathematical dialogue, metacognitive coaching, and relationship-building. However, AI may redistribute rather than reduce teachers’ workload: verifying outputs, monitoring dashboards, managing errors and handling technical failures create new tasks, and the net effect on teacher time is an empirical question that current evidence does not settle. Adaptive platforms may also help teachers systematically adjust the difficulty and pace of whole-class instruction, so more learners are appropriately challenged without the teacher having to track and manage each pathway individually. Appendix A indicates the type of evidence behind each of these claims.

The meta-analytic evidence on intelligent tutoring in schools is also more mixed than the opportunities above might suggest. Synthesising 26 reports (34 independent samples) of ITS use in K–12 mathematics, Steenbergen-Hu and Cooper (2013) found average effects ranging from only *g* = 0.01 to *g* = 0.09, with smaller benefits for low achievers than for the general student population, and apparently larger effects for interventions lasting less than a school year. Across 50 evaluations, Kulik and Fletcher (2016) report a median gain of 0.66 standard deviations, but much smaller gains when outcomes were measured on standardised rather than locally developed tests. Ma et al. (2014) found that ITSs outperformed teacher-led large-group instruction (*g* = 0.42) but held no significant advantage over individual human tutoring or small-group instruction. Few of these studies isolate the primary years, and none tests the role of teacher mediation directly. Small, heterogeneous and sometimes null effects are, however, what a teacher-mediation account predicts: if the benefits of AI depend on orchestration, scaffolding quality and contextual conditions (P3–P5), effects should vary as those conditions vary. The mixed evidence therefore motivates, rather than weakens, the framework’s central proposition.

### 7.2 Challenges

Teacher readiness is the first and most fundamental obstacle. Available evidence suggests that many teachers lack the skills to use AI tools in a pedagogically sound, critically informed way. Weak AI-TPACK knowledge, stemming from limited professional development, exactly the gap ODeL professional development is meant to close (Li, 2025; Ng et al., 2023), can lead to AI being misused or underused, with neutral or even harmful effects on learning.

The digital divide is a second, structural challenge. It is easy to assume AI tools are broadly available, but that assumption conceals real disparities in access, particularly for teachers and learners in low-income or rural areas without reliable internet, adequate devices, or stable power (Faridi and Shaheen, 2024; Mhlongo et al., 2024). An unreliable electricity supply limits teachers’ participation in ODeL programmes and restricts learners’ access to AI-based mathematics tools, resulting in both groups losing out on the potential benefits of human–AI collaboration. Meeting the equity commitments of Sustainable Development Goal 4 will require policymakers and ODeL programme designers to treat infrastructure and energy access as part of the problem, not an afterthought, particularly in low-income communities (Cukurova and Miao, 2024).

Even well-resourced settings face a third challenge: over-reliance. Some learners will treat AI as a shortcut to answers rather than a partner in reasoning, bypassing the productive struggle that builds real mathematical understanding; analogical evidence from secondary mathematics shows that unrestricted generative AI can depress later unassisted performance (Bastani et al., 2025). ODeL programmes that fail to address this directly are unlikely to produce the kind of deep professional learning that authentic human–AI collaboration requires. Teachers need to actively set and uphold standards for appropriate AI use, helping learners distinguish between AI-assisted thinking and AI-substituted thinking by building their metacognitive awareness (Table 4).

Finally, ethics, privacy, and bias remain open and serious questions. As AI systems accumulate large volumes of learner data, concerns about privacy, consent, and algorithmic bias grow with them, especially where tools are trained predominantly on data from affluent, English-speaking contexts, which risks disadvantaging learners from other linguistic and socio-economic backgrounds (Holmes et al., 2022). Teachers need to develop ethical judgement in tool selection, familiarity with relevant data protection rules, and a critical eye to spot cultural and linguistic bias in AI tools, all of which require deliberate attention within ODeL professional development, rather than assuming they will be picked up incidentally. For primary learners, however, these questions extend further and are treated in Section 7.3 as a boundary condition rather than as one challenge among others.

### 7.3 Governance and children’s rights as a cross-cutting boundary condition

For primary learners, the ethical questions determine whether the proposed collaboration is acceptable at all. The framework, therefore, treats governance as a boundary condition that cuts across all three layers (Figure 1; P7). Drawing on children’s rights guidance for AI and the digital environment (UNICEF, 2021; United Nations Committee on the Rights of the Child, 2021) and, for the primary context, South Africa’s Protection of Personal Information Act (Republic of South Africa, 2013), eight requirements are specified.

#### 7.3.1 Parental consent and child assent

Processing children’s personal information generally requires the consent of a competent person, usually a parent or guardian; children should in addition receive an age-appropriate explanation and the opportunity to assent or decline.

#### 7.3.2 Data minimisation and retention

Only data necessary for pedagogical purposes should be collected, retention periods should be defined, and data should be deleted when the purpose has lapsed.

#### 7.3.3 Automated profiling

Learner models and risk flags should not be used to make consequential decisions about a child, such as long-term ability grouping or promotion, without human review.

#### 7.3.4 Explainability and the right to contest

Teachers, parents and, in age-appropriate ways, learners should be able to understand why a system recommended a task or flagged a learner, and to contest that inference.

#### 7.3.5 Child safeguarding

Conversational tools should be restricted to curriculum purposes and filtered for harmful content, young children should not use them unsupervised, and transcripts should be reviewed.

#### 7.3.6 Commercial use of data

Learner data should not be used for advertising, sold, or used to train commercial models without explicit, informed agreement, and procurement contracts should say so.

#### 7.3.7 Accessibility and disability

Tools should meet accessibility standards and support assistive technologies, so that learners with disabilities are neither excluded nor misclassified by learner models.

#### 7.3.8 Linguistic fairness

In multilingual classrooms, tools should support learners’ home languages where possible, and teachers should check whether apparent mathematical difficulty reflects language demand—a particular concern where the language of learning and teaching changes in the Intermediate Phase.

These requirements shape each layer. In Layer 1, they define the ethics component of AI-TPACK and the governance component of ODeL programmes; in Layer 2, they constrain which tools may be selected and how they are configured; in Layer 3, they determine which learner data may be collected as evidence. Where they cannot be met, the framework does not recommend AI use, however promising the pedagogical case.

## 8 Implications

The framework developed above has practical implications for five groups of stakeholders. Table 7 summarises the priority actions for each, links each to the framework’s constructs and propositions, and proposes implementation and evaluation indicators; the discussion below expands on the reasoning behind them.

**Table 7:** Priority actions, linked constructs and propositions, and implementation and evaluation indicators, by stakeholder group.

Stakeholder	Priority actions	Linked construct/proposition	Implementation and evaluation indicators
Teacher education institutions	Design ODeL programmes on the five principles (Section 4.5), targeting all five AI-TPACK components; provide mentoring and formal recognition	ODeL professional development; AI-TPACK; P1, P5	Dosage (hours, practice cycles, completion by group); design quality (fidelity to principles, facilitator dialogue); pre– and post–change in AI-TPACK (scale and rubric-scored artefacts)
Policymakers	Set teacher AI competency standards aligned with UNESCO’s framework; fund ODeL at scale; enforce data protection and equity of access	Governance (P7); infrastructure moderator (P5)	Share of funded teachers in rural and low-fee schools; participation and completion gaps; compliance audits against data protection law
School leaders	Protect time for collaborative learning; set school norms for AI use; invest in connectivity, devices and electricity; oversee consent and data governance	Orchestration (P2, P3); school support and infrastructure moderators; governance (P7)	Orchestration fidelity (observation checklist); working device and connectivity ratios; consent and assent records
Researchers	Test P1–P7 through multilevel longitudinal, design-based and comparative (ODeL versus blended versus face-to-face) studies	All propositions	Tests of mediation (P3) and moderation (P5); quality of mathematical reasoning; AI dependence; disparities in outcomes by gender, home language, disability and school type
Learners (rights holders)	Take part in selecting, evaluating and rejecting tools; learn productive ways of working with AI	Learner outcomes; AI dependence; governance (P7); F2	Existence and use of learner-voice mechanisms; learner-reported experience; dependence indicators (Table 4); disparities in outcomes across groups

### 8.1 Teacher education institutions

ODeL institutions should build AI-focused professional development programmes for practising primary mathematics teachers. Such programmes should be organised around the five components of AI-TPACK (Section 4.3); apply the five ODeL design principles derived in Section 4.5, of sustained engagement, practice-based learning, collaborative community, contextual responsiveness, and congruent modelling; combine foundational theory with hands-on experimentation; and build in ongoing mentoring, peer collaboration, and reflective practice. Formal recognition, such as continuing professional development credit within national accreditation systems, would help sustain participation among teachers juggling competing professional demands. Programmes should monitor dosage and design quality and evaluate change in AI-TPACK using both self-report and performance-based indicators, as P1 requires.

### 8.2 Policymakers

National and regional policymakers should articulate AI competency standards for teachers, spelling out the knowledge, skills, and dispositions effective human–AI collaboration in primary mathematics requires, standards that can then inform professional development design, teacher performance frameworks, and pre-service curricula. Policy should also fund sustained ODeL-delivered professional development for AI integration; buying technology without investing in teacher preparation is unlikely to accomplish much. Equity of access needs to be an explicit policy commitment so that low-income and rural schools are not locked out of AI-enhanced mathematics instruction due to infrastructure gaps, and regulatory frameworks should protect learners’ data privacy and hold AI tools accountable, giving effect to the governance conditions in Section 7.3 (P7).

### 8.3 Schools

School leaders create the institutional conditions that let teachers apply what ODeL professional development has given them. That means protecting time and resources for ongoing collaborative professional learning, setting clear school-level norms for appropriate AI use, investing in the connectivity, devices, and stable electricity AI-enhanced instruction depends on, and cultivating a school culture of critical, reflective engagement with educational technology, neither uncritical adoption nor reflexive rejection. Leaders should also support teachers in navigating data privacy obligations and ensuring AI use complies with relevant legislation and ethical guidelines. Because school support is proposed to moderate the translation of AI-TPACK into orchestration (P2, P3), leaders can monitor orchestration fidelity as an indicator of whether these conditions are in place.

### 8.4 Researchers

The framework proposed here is conceptual and needs empirical testing. Its propositions (Table 5) specify what should be tested. Several directions stand out as priorities. Design-based research should develop, implement, and iteratively refine ODeL-based professional development programmes for AI integration in primary mathematics, using the three-layer framework as the starting point for design. Longitudinal multilevel studies should track how teachers’ AI-TPACK develops through ODeL programmes and how that development translates into classroom practice and learner outcomes, testing whether orchestration mediates the link (P3) and whether the proposed moderators operate (P5); comparative studies should set ODeL against blended and face-to-face professional development. Sociolinguistic and ethnographic research should examine the social dynamics of AI-mediated primary mathematics classrooms, how learners move between AI interaction and peer mathematical dialogue, and how teachers manage that movement in practice. Legal and ethical research should examine how AI tools align with data protection frameworks and the equity implications of algorithmic systems operating in primary mathematics education. Across these designs, outcome disparities across groups of learners should be reported routinely.

### 8.5 Learners

If the framework’s propositions hold, human–AI collaboration may offer primary learners a qualitatively different, more equitable mathematics learning experience. Individualised scaffolding, adaptive feedback, and tailored problem-solving support, once available mainly to learners with access to private tuition, can, in principle, become standard classroom practice. Learners in under-resourced communities, who are at greater risk of falling behind without targeted support, could gain the most from well-coordinated AI tools, in line with the Sustainable Development Goal 4 commitment to inclusive, equitable, quality education for all—but they are also the most exposed to infrastructure gaps and to tools not designed for their languages.

That said, learners need deliberate instruction on how to work well with AI. Left unguided, they may drift into dependent, answer-seeking interactions with AI systems rather than developing the self-regulated, metacognitively aware problem-solving that primary mathematics education is meant to build. Teachers need to actively cultivate learners’ understanding of AI as a thinking partner, not just an answer source, by building classroom norms that treat AI interaction as work requiring the learner’s own effort, reasoning, and reflection (Kapur, 2016). And learners in energy-poor, digitally marginalised communities face a compounding disadvantage: the same infrastructure gaps that limit their teachers’ access to ODeL professional development also cut them off from AI-enhanced mathematics education, widening an already worrying divide. Addressing that intersection of energy poverty, digital exclusion, and mathematics underachievement will take coordinated effort across education policy, technology design, and infrastructure investment (Cukurova and Miao, 2024).

Learners should also be positioned not only as beneficiaries but as rights holders with agency and voice (United Nations Committee on the Rights of the Child, 2021; UNICEF, 2021). In practice, this means age-appropriate explanations of what AI tools do and what happens to their data, the opportunity to assent or decline, classroom routines in which learners judge whether a tool helped their thinking, and channels through which their views inform the selection, continued use or rejection of technologies. Learners’ accounts of their experience are thus also evidence (F2) for teachers and programme designers.

## 9 Discussion

This paper set out to answer a conceptual question: through which mechanisms, and under which conditions, can ODeL-based professional development be expected to enable teacher-facilitated human–AI collaboration that supports primary learners’ mathematical problem-solving? The answer developed across the preceding sections rests on treating the teacher consistently as an orchestrator of AI rather than a bystander, a framing with implications worth drawing out explicitly.

The three-layer framework proposed here departs from two more common ways of talking about AI in education. It is not a technology-adoption narrative, in which better tools straightforwardly produce better outcomes once teachers are trained to operate them. And it is not a replacement narrative, in which AI’s growing sophistication is treated as evidence that the teacher’s role will shrink over time. Instead, the framework treats teacher expertise and AI capability as genuinely complementary, each compensating for something the other cannot do, and makes the strength of that complementarity, rather than the sophistication of the technology alone, the thing worth optimising for. This position is consistent with arguments that AI benefits depend on both teacher competence and tool quality (Celik, 2023; Eti et al., 2026; Holstein et al., 2019), although, as noted, direct evidence for primary mathematics remains limited.

### 9.1 Comparison with existing frameworks

Table 8 compares the framework with the models it builds on.

**Table 8:** Comparison with existing frameworks.

Framework	Primary focus and unit	What it explains	What it leaves open	What this framework adds
AI-TPACK/Intelligent-TPACK (Celik, 2023; Mishra et al., 2023)	Teacher knowledge; the teacher	The knowledge components needed to integrate AI ethically, and how to measure them	How the knowledge develops through professional development; how it becomes classroom practice	Programme-level mechanisms (P1); enactment through orchestration (P2–P3)
Classroom orchestration (Dillenbourg, 2013)	Teacher coordination of multi-plane activity; the classroom	The constraints on, and design of, teachers’ management of classroom activity	AI-specific verification, fading and governance; how orchestration is learned	Orchestration as mediator between AI-TPACK and scaffolding quality (P3); scaffolding criteria
Teacher–AI complementarity and hybrid adaptivity (Holstein and Aleven, 2022; Holstein et al., 2019, 2020; Molenaar, 2022)	Division of adaptive work between teacher and AI; classroom and interaction	How adaptivity and control can be shared; levels of automation from teacher-only to full automation	Teacher preparation; mostly well-resourced settings and older learners	Professional development pathway; moderators (infrastructure, age, language); F1/F2 distinction
Teacher-in-the-loop and human-centred AI (EdTech Hub, 2025; Shneiderman, 2020)	Human control over automated systems; system design	Why high automation and strong human control can coexist; design principles	Pedagogical criteria for oversight in mathematics; how teachers acquire the capacity	What “in the loop” means pedagogically (validation, fading, Polya-phase criteria); children’s-rights governance
UNESCO AI competency framework for teachers (Cukurova and Miao, 2024)	Competencies and progression; teacher and system	What teachers should be able to do across five dimensions and three levels	Mechanisms linking competence to learner outcomes	A propositional causal chain with indicators

The comparison with levels-of-automation accounts is the most important. Molenaar (2022) formalises how control over monitoring, diagnosis, and intervention is distributed between teacher and technology, from teacher-only to fully automated instruction, and Holstein and Aleven (2022) set out how the strengths of teacher and AI can complement one another in K–12 classrooms. These models describe what a given division of control looks like; the present framework adds three things they do not treat. First, it provides a developmental account of how teachers become able to operate at a given level of automation through ODeL professional development that builds AI-TPACK (P1–P2), a problem that the orchestration literature has largely left aside by assuming a capable teacher. Second, it supplies pedagogical criteria contingency, fading and transfer of responsibility (P3–P4) for judging whether a given division of control produces good scaffolding. Third, it specifies conditions infrastructure, learner age, language and governance (P5, P7) under which higher levels of automation are or are not appropriate for primary learners. In levels-of-automation terms, the framework predicts that the appropriate level is not fixed by the technology but depends on teacher capacity and learner age, and that moving young learners toward higher automation without the corresponding orchestration capacity will lower scaffolding quality.

Three elements are, as far as I can determine, new: (1) the specification of transactional-distance mechanisms through which ODeL professional development is expected to build AI-TPACK; (2) the positioning of teacher orchestration as a mediator between AI-TPACK and scaffolding quality, with scaffolding quality defined by contingency, fading and transfer of responsibility; and (3) the treatment of governance and children’s rights as a boundary condition within the framework. Two relationships are revised. The implicit assumption in AI-TPACK work that knowledge translates directly into practice is replaced by a mediated relationship (P3), and the assumption in teacher-in-the-loop models that the teacher is already capable is replaced by a developmental pathway (P1–P2). The framework thereby offers a candidate explanation for a pattern that existing models leave unresolved: why similar AI tools appear to yield different results across classrooms and contexts. It attributes this variation to differences in orchestration, scaffolding quality and moderating conditions.

### 9.2 Alternative explanations, Equifinality and falsification

Alternative explanations must be taken seriously. Improvements observed after ODeL professional development might reflect general pedagogical quality teachers who orchestrate AI well may simply be better teachers rather than AI-specific capacity; they might reflect school-level support, such as leadership, resources or a collaborative culture; or they might reflect selection, because teachers who enrol in and complete ODeL programmes may differ systematically from those who do not. The framework also allows for equifinality: similar learner outcomes may be reached by different routes, such as high-quality teaching without AI or well-designed AI with modest teacher involvement for older learners. Tests of the framework should therefore control for baseline pedagogical quality and school-level support, compare ODeL with blended and face-to-face professional development, and include non-AI comparison conditions.

The framework’s propositions would be weakened or falsified if (a) ODeL programmes meeting the five design principles with adequate dosage produced no measurable growth in AI-TPACK relative to comparison groups (P1); (b) growth in AI-TPACK did not predict orchestration quality (P2); (c) orchestration did not mediate the relation between AI-TPACK and scaffolding quality, for example because AI-TPACK affected scaffolding only through tool selection (P3); (d) learners in classes with higher scaffolding quality showed no advantage in unassisted reasoning, or no lower AI dependence, than learners with equivalent AI access but lower scaffolding quality (P4); (e) the predicted moderators showed no moderating effects (P5); or (f) learner gains were fully explained by general pedagogical quality or school-level support once these were controlled.

### 9.3 Limitations

The framework also has real limits, which are worth stating plainly rather than glossing over. First, it is a conceptual synthesis, not an empirically validated model; the relationships it proposes among layers are theoretically motivated but have not yet been tested against classroom data, and Section 8 sets out the empirical agenda needed to do so. Second, the framework was developed with reference to a particular context, primary mathematics in South Africa, ODeL-based teacher development, and resource-constrained settings, and its transferability to other countries, subjects, phases of schooling, or well-resourced contexts is an open question rather than an assumption. Third, although learner age and language are now specified as moderators (P5), the framework is less developed on other learner-level variation: individual differences in prior AI exposure, disability, or motivation likely shape how any given learner experiences AI scaffolding, and a fuller account would need to account for that variation rather than treat learners as a relatively uniform Layer 3. Fourth, because this integrative review was conducted with support from an AI discovery tool, the evidence base may be incomplete despite the verification procedure outlined in Section 2.2.

There is also a tension that the paper has tried not to paper over. AI’s core educational promise, personalisation at a scale no teacher can match, is also where its risk is concentrated: over-reliance, algorithmic bias, and data practices that primary teachers are rarely equipped to scrutinise. The framework’s answer is that teacher orchestration is the mechanism that keeps this promise from tipping into risk, but that answer is only as good as the professional development that produces orchestration capacity in the first place. If ODeL programmes fail to build AI-TPACK, the framework offers no fallback, which is precisely why Section 7 treats teacher readiness as the first and most consequential challenge rather than a minor implementation detail.

Read together, these limitations point toward a specific research agenda: testing whether the framework’s proposed relationships and feedback loops operate as described in real ODeL programmes and real classrooms, and doing so across a wider range of contexts than the one this paper has focused on.

## 10 Conclusion

This paper has examined whether ODeL-based professional development can prepare primary school teachers for genuine human–AI collaboration in mathematics instruction, thereby strengthening learner problem-solving. Rather than restating Section 5, the conclusion consolidates what the argument adds. Teacher professional development is proposed not as an optional add-on to AI adoption but as an enabling condition for its benefits. However sophisticated AI tools become, without teachers equipped with integrated AI-TPACK knowledge to use them deliberately, critically, and ethically, those tools are hypothesised to be unlikely to produce durable improvements in learners’ mathematical problem-solving. ODeL is proposed to make this development reachable for teachers across very different geographic and socio-economic contexts, if participation conditions are met, and programmes are built around the five design principles derived in Section 4.5: sustained engagement, practice-based learning, collaborative community, contextual responsiveness, and congruent modelling as design essentials, not afterthoughts, and backed by policy investment that addresses digital access gaps and infrastructural inequity.

AI is proposed to function best as part of a teacher AI system that provides contingent, fading scaffolding within learners’ ZPD, extending teachers’ capabilities rather than replacing them. The theoretical analysis here suggests that AI tools offer pedagogically relevant strengths in personalised scaffolding, adaptive feedback, and learning analytics that may improve the reach and quality of primary mathematics instruction if model accuracy, task design, and orchestration are adequate. But those strengths are bounded: AI lacks the genuine intersubjectivity, cultural sensitivity, emotional attunement, and ethical judgement that define good human teaching.

Bringing together Vygotsky’s ZPD, social constructivism, Intelligent-TPACK, and Polya’s four-phase model within a multilevel meta-model offers a theoretically coherent foundation for teacher-facilitated human–AI collaboration in primary mathematics. The three-layer framework of professional development and teacher capacity, teacher-orchestrated AI scaffolding, and learner problem-solving offers teacher educators, policymakers, school leaders, and researchers a shared, theoretically grounded reference point for programmes, policies, and research aimed at strengthening primary learners’ mathematical problem-solving through principled human–AI collaboration. In the end, the real measure of success here is not how advanced the AI tools become, or how many professional development initiatives are launched, but whether primary learners in AI-enhanced classrooms, taught by well-prepared, critically engaged teachers, learn more mathematics.

A conceptual analysis cannot support conclusions about effectiveness, so the article ends instead with testable propositions and their boundary conditions: that ODeL professional development of adequate design and dosage builds AI-TPACK (P1); that AI-TPACK shapes scaffolding quality through teacher orchestration (P2–P3); that scaffolding quality, rather than AI access alone, predicts learners’ reasoning and lower AI dependence (P4); that these relationships are moderated by infrastructure readiness, learner age, language and AI type (P5) and strengthened when teachers triangulate system-generated and observed evidence (P6); and that none of them applies where governance conditions protecting children’s rights are unmet (P7). Testing these propositions in South African primary classrooms and examining whether they transfer to other contexts is the next step.
