# Supplementary material for: An ODeL-based teacher professional development framework for teacher-facilitated human–AI collaboration in primary mathematics problem-solving: a conceptual analysis
Source: Front Artif Intell. 2026 Oct 23;9:1970792. doi: 10.3389/frai.2026.1970792 (PMC13739525; doi:10.3389/frai.2026.1970792)
Supplement: Supplementary file 1 [file Supplementary_file_1.docx]

**Zanele Dibane**

**Appendix A. Evidence of Sources Cited for Substantive Claims**

Evidence type: D = direct (empirical, primary mathematics); A = analogical (empirical, other level, subject or population); T = theoretical, conceptual or review without new data; G = grey literature (policy, report, blog).

| **Source** | **Type** | **Level and context** | **Used for** | **Limits on transfer to primary mathematics** |
| --- | --- | --- | --- | --- |
| Abinaya et al. (2024) | T | Book chapter on ITSs, all levels | ITS definition: feedback | Not specific to primary mathematics |
| Baidoo-Anu & Owusu Ansah (2023) | T | Review of ChatGPT in teaching and learning | Chatbot risks | Not child-specific; risk for young learners extrapolated |
| Bastani et al. (2025) | A | Field experiment, high school mathematics | Over-reliance on generative AI | Older learners; different curriculum and context |
| Celik (2023) | A / T | Survey and scale development with teachers | AI-TPACK construct and measurement | Sample not specific to primary mathematics |
| Crompton & Burke (2023) | A | Systematic review, higher education | Teacher-competence argument | Higher education; transfer by analogy only |
| Cukurova & Miao (2024) | G | UNESCO policy framework | Competency dimensions; equity | Normative, not empirical |
| Darling-Hammond et al. (2017) | A / G | Research review (report), mainly face-to-face professional development | Features of effective professional development | Not AI-specific; limited distance-mode evidence |
| Dignath et al. (2008) | D | Meta-analysis of self- regulation training in primary schools | Teachability of  self- regulation at primary age | Not AI-specific |
| EdTech Hub (2025) | G | Blog post summarising an expert panel | Failure modes of AI use | Expert opinion; not peer reviewed |
| Eti et al. (2026) | A / D | Systematic review of 20 studies, primary to tertiary | Teacher mediation; dependency risk | Mixed levels; primary-specific findings not separated |
| Graven & Pausigere (2017) | D | In-service community of practice, South African primary mathematics | Professional community; social constructivism | Not AI-specific |
| Hamedi et al. (2025) | T | Integrative review, education generally | AI as ZPD scaffold | Not mathematics- or primary-specific |
| Holmes et al. (2022) | T | Community-wide ethics framework | Ethics, bias | Not empirical |
| Holstein et al. (2019); Holstein & Aleven (2022) | A / T | Design research on teacher–AI complementarity, K–12 (mainly middle school) | Orchestration and complementarity | Older learners; well-resourced settings |
| Hwang & Tu (2021) | T | Bibliometric review, mathematics education, all levels | Research trends | Maps trends; does not test effects |
| Kasneci et al. (2023) | T | Commentary on large language models, all levels | Chatbot capabilities; analytics | Not primary- or mathematics-specific |
| Kulik & Fletcher (2016) | A | Meta-analysis of 50 ITS evaluations, mixed levels | Mixed ITS effects; test alignment | Mostly older learners |
| Li (2025) | D | Primary mathematics teachers’ AI adoption | Teacher readiness | Adoption rather than classroom outcomes |
| Liu et al. (2025) | D | Design and evaluation study, primary mathematics word problems | Chatbot scaffolding | Single study; generalisability unknown |
| Ma et al. (2014) | A | Meta-analysis of ITS outcomes across levels and subjects | ITS versus other instruction | Not primary-specific |
| Mhlongo et al. (2024) | D / A | South African mathematics teachers, online resources, context of disadvantage | ODeL access; infrastructure | Not AI-specific |
| B. A. Moore et al. (2024) | A | Online professional development, rural middle-school science | Online professional development effectiveness | Different subject and level |
| Ng et al. (2023) | A | Teachers’ AI digital competencies | Teacher AI competencies | Not specific to primary mathematics |
| Polater & Çelik (2026) | D | Systematic review of 24 ITS studies, primary mathematics | ITS effects; teacher training needs | Concentrated on number and operations; implementation-dependent |
| Steenbergen-Hu & Cooper (2013) | D / A | Meta-analysis, ITSs in K–12 mathematics (26 reports) | Mixed ITS effects at school level | Primary years not isolated |
| Thiele & Bogdon (2022) | A | Virtual professional learning network, rural STEM teachers | Distance professional community | Not primary- or AI-specific |
| VanLehn (2011) | A | Review of tutoring studies, mainly secondary and post-secondary | ITS effectiveness; feedback granularity | Older learners; pre-generative AI |
| Venkat & Spaull (2015) | D | South African primary teachers’ content knowledge (SACMEQ) | Context; content knowledge | Not AI-specific |
| Whitebread et al. (2009) | D | Observational studies of young children’s metacognition | Developmental grounding of Polya’s phases | Early years; not mathematics- or AI-specific |
| Xie et al. (2019) | A | Systematic review of adaptive learning, all levels | Adaptive feedback | Not primary-specific |
